# The Treatment of High Degrees of Myopia

**Published:** 1899-12-02

**Authors:** 


### THE TREATMENT OF HIGH DEGREES OF MYOPIA.

In a paper recently read before the Leeds and West Riding Medico-Chirurgical Society, Dr. Adolph Bronner said that one of the most interesting and important advances in ophthalmic surgery during the last few years had been the removal of the lens in cases of high myopia. In regard to the choice of cases he thought that we ought to operate in all cases of myopia in which, with the help of concave glasses, the patient cannot obtain such an amount of vision as to enable him to follow any useful occupation, or in which the use of glasses, although affording serviceable vision, causes severe pain or discomfort. The great objection to the removal of the lens is the possibility of consequent detachment of the retina, which again may to some extent depend upon the degree of skill with which the operation is performed. Then there is the risk of septic inflammation, or glaucoma may set in, or there may be iritis, or anterior or posterior synechiæ. "It is therefore ridiculous to say that the operation is quite harmless and not attended by any great risk, as some surgeons assert." On the other hand, a high degree of myopia is itself attended with risk to the eye and is apt to increase, so that in removing the lens in such cases the operation is done not only with the object of procuring better and more serviceable vision but of preventing increase of the myopia.

